# Interleukin 1 β suppresses bile acid-induced BSEP expression via a CXCR2-dependent feedback mechanism

**DOI:** 10.1371/journal.pone.0315243

**Published:** 2024-12-16

**Authors:** Carolin Angendohr, Leah Missing, Christian Ehlting, Stephanie D. Wolf, Karl S. Lang, Mihael Vucur, Tom Luedde, Johannes G. Bode

**Affiliations:** 1 Faculty of Medicine & Düsseldorf University Hospital, Department of Gastroenterology, Hepatology and Infectious Disease, Heinrich-Heine-University, Düsseldorf, Germany; 2 Department of Immunology, University of Essen, Essen, Germany; University of Navarra School of Medicine and Center for Applied Medical Research (CIMA), SPAIN

## Abstract

Inflammation-induced cholestasis is a common problem in septic patients and results from cytokine-mediated inhibition of bile acid export including impaired expression of the bile salt export pump (BSEP) with a consecutive increase in intracellular bile acids mediating cell damage. The present study focuses on the mechanisms by which interleukin 1 β (IL-1β), as a critical mediator of sepsis-induced cholestasis, controls the expression of BSEP in hepatocytes. Notably, the treatment of hepatocytes with IL-1β leads to the upregulation of a broad chemokine pattern. Thereby, the IL-1β -induced expression of in particular the CXCR2 ligands CXCL1 and 2 is further enhanced by bile acids, whereas the FXR-mediated upregulation of BSEP induced by bile acids is inhibited by IL-1β. In this context, it is interesting to note that inhibitor studies indicate that IL-1β mediates its inhibitory effects on bile acid-induced expression of BSEP indirectly via CXCR2 ligands. Consistently, inhibition of CXCR2 with the inhibitor SB225002 significantly attenuated of the inhibitory effect of IL-1β on BSEP expression. These data suggest that part of the cholestasis-inducing effect of IL-1β is mediated via a CXCR2-dependent feedback mechanism.

## Introduction

The inflammation-induced cholestasis is a frequent problem in intensive care unit. Approximately 20% of patients suffering from systemic inflammation have aspects of cholestasis without any known prior liver disease [1,2]. The state of an overwhelming inflammation with consecutive organ damage is defined as sepsis and since 2017 the elevation of bilirubin as a parameter of impaired bile flow is part of the sepsis definition [3,4].

Since the discovery of cholestasis in septic patients in the 1960s and 1970s great advances have been made in the pathophysiology of sepsis induced cholestasis [5,6]. The first milestone was set when studies revealed that the pathogen-associated molecule lipopolysaccharide (LPS), a cell wall component released by Gram-negative bacteria, is responsible for the inflammation-induced cholestasis [7,8].

Further studies have shown that the development of cholestasis in response to LPS treatment is largely due to increased production of inflammatory cytokines, particularly IL-1β and tumour necrosis factor-α (TNF-α). Accordingly, blocking the action of IL-1β or TNF-α with an antibody directed against the IL-1β receptor or a TNF-α inactivating fusion protein results in a reduction of LPS-induced cholestasis [8–12], which was almost completely abolished by combined inactivation of IL-1β and TNF-α [13]. The production of the latter cytokines mainly occurs by cell populations such as macrophages in particular, but also by other non-parenchymal cells such as endothelial cells or hepatic stellate cells. On the other hand, the vast majority of existing studies suggest that hepatocytes are a potential source of chemotactic signals, but play a rather subordinate role with regard to the synthesis of cytokines such as TNF-α, IL-6 or IL-1β [14–17].

While one effect of inflammation-related organ damage is due to exacerbated cytokine-mediated infiltration of immune cells into the end organ with subsequent tissue damage, cytokines also act directly on hepatocytes and interfere with the molecular mechanisms of bile acid regulation. In this regard, dysregulation of the localization, protein levels and transcript expression of proteins in bile salt transport are essential mechanisms by which LPS and inflammatory cytokines such as TNF-α and IL-1β lead to the development of cholestasis.

The bile salt export pump BSEP, which is located at the apical membrane of the hepatocyte, plays a central role in the transport of bile salts from the hepatocyte lumen into the canaliculi. Consequently, BSEP is expressed only in hepatocytes and not in any other cell type of the organism because this function is performed exclusively in hepatocytes [18].

BSEP transports bile acids such as deoxycholic acid, conjugated cholic acid, urodeoxycholic acids, and chenodeoxycholic acid (CDCA), with CDCA being the major substrate [19–21]. A clinical entity that highlights the importance of BSEP is progressive familial intrahepatic cholestasis (PFIC)-2, which is due to the absence of BSEP at the biliary canalicular membrane and leads to the development of cirrhosis and hepatocellular carcinoma in early childhood [22,23].

In the hepatocyte the protein content of BSEP is regulated by transcriptional and post-transcriptional mechanisms, which are leadingly controlled by the availability of bile acids themselves, ensuring a closed adaptation to physiological and pathophysiological conditions [24,25]. The transcription factor farnesoid X receptor (FXR), that is activated in response to rising concentrations of distinct bile acids such as CDCA [26–28] plays a key role in the transcriptional control of BSEP expression. This is mediated by heterodimer formation of FXR with the retinoid X receptor (RXR), which like FXR belongs to the family of nuclear receptors [28–30]. Apart from BSEP, FXR also mediates the expression of the transcription factor SHP [31], which, although lacking a DNA-binding domain, is involved as a transcription factor in the transcriptional control of various transport proteins including BSEP, representing a kind of feedback control [32].

It is well known that in sepsis-induced cholestasis, the mechanisms that ensure adaptation to bile acid concentration are impaired and BSEP expression is decreased [33,34]. The precise molecular mechanisms underlying deregulation of BSEP expression by inflammatory cytokines are poorly understood to date.

This is especially true with regard to possible feedback mechanisms conveyed by mediators whose expression is induced by inflammation in hepatocytes. Thus, it is known that in hepatocytes, IL-1β, among others, is a potent elicitor of chemokine expression such as CXCR2 ligands, which may be involved in mediating the inhibitory effect of IL-1β on *Bsep* gene expression. The present manuscript investigates the potential engagement of chemokines in mediating the inhibitory effects of particularly IL-1β in the context of LPS-induced inflammation on inducible *Bsep* gene expression in hepatocytes.

## Results

### Already low concentrations of IL-1β or TNF-α are sufficient to impair CDCA-induced BSEP gene expression in murine hepatocytes and HepaRG cells

**Fig 1:**
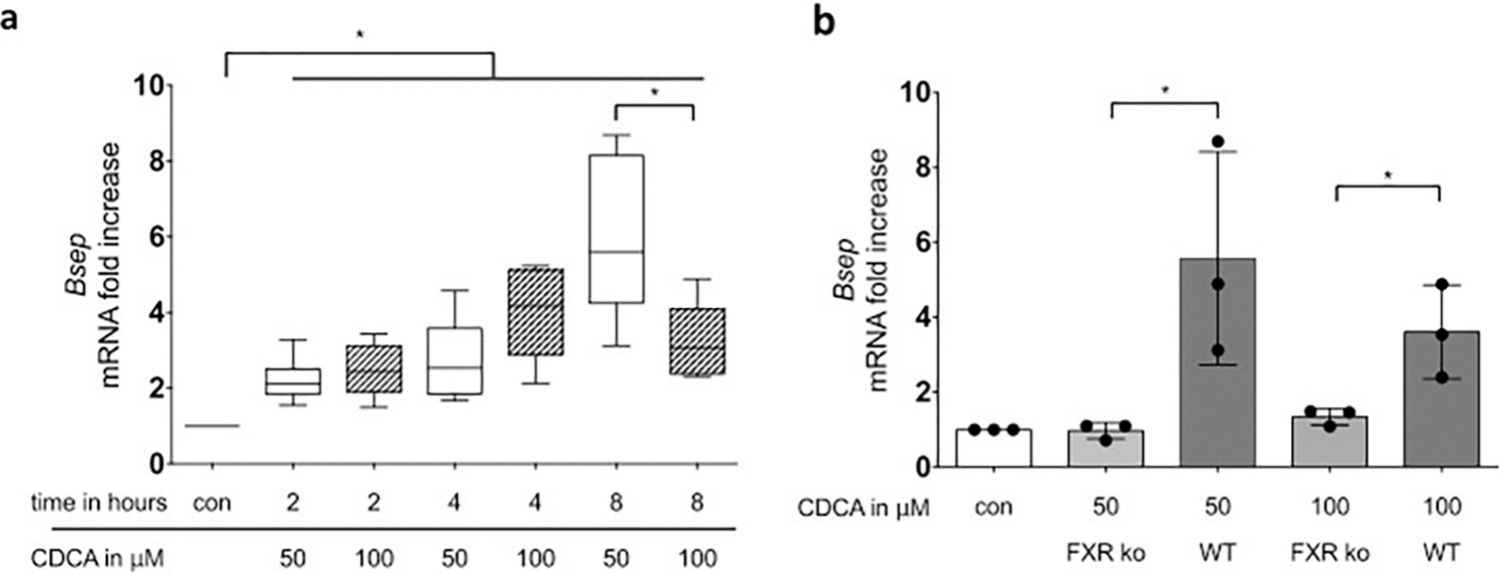
CDCA induces *Bsep*-expression in a time and concentration dependent manner which is dependent on FXR. Primary mouse hepatocytes from WT animals (**a**) were cultivated in sandwich culture and stimulated with the indicated concentrations of CDCA. WT and FXR-/- (**b**) animals were cultivated in sandwich culture and stimulated with the indicated concentrations of CDCA for eight hours. After the respective time period RNA was prepared and transcript abundance was determined by qRT-PCR using primers specific for BSEP. Data are presented as box blots based on 3 to 6 replicates. Statistics were calculated by Whitney U test for **a** and t-test for **b**. A p-value of less than 0.05 was considered significant (* p<0.05).

To study the effects of inflammatory cytokines on inducible *Bsep* expression in PMH, a culture model was chosen that allows the study of primary hepatocytes under steroid-free conditions without eliciting epithelial to mesenchymal transition of these cells. Under these conditions, treatment with 50 μM CDCA, the strongest inducer of Bsep gene expression [35,36], for 8 hours induced FXR-dependent Bsep gene expression in primary murine hepatocytes (Fig 1A and 1B). As shown by cell viability testing using colourimetric determination of formazan formation, cell viability is not limited at this concentration, while a further increase to 100 μM leads to a time-dependent decrease in cell viability (S1 Fig in S1 File), which is non-toxic.

**Fig 2:**
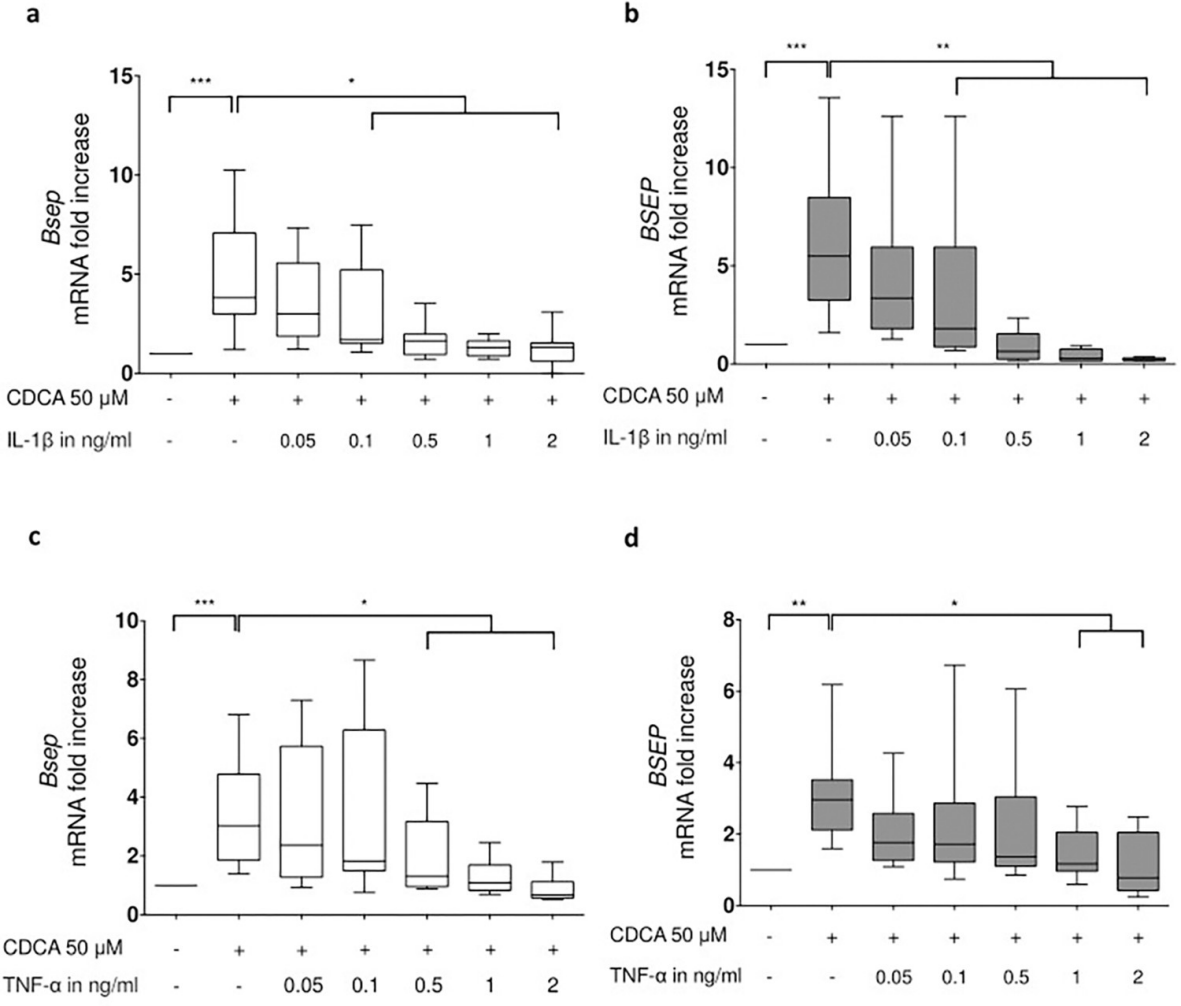
IL-1β and TNF-α suppress the CDCA induced BSEP expression in a concentration dependent manner in PMH and HepaRG cells. PMH (**a**, **c**) and HepaRG cells (**b**, **d**) were preincubated with the indicated concentrations of IL-1β (**a**,**b**) or TNF-α (**c**,**d**) for 60 minutes or left untreated for control and subsequently stimulated with 50 μM CDCA for 8 hours. Thereafter total RNA was prepared and transcript abundance was determined by qRT-PCR using primers specific for BSEP. Data are presented as box blots based on 4 to 8 replicates. Statistics were calculated by Whitney U test. A p-value of less than 0.05 was considered significant (* p<0.05; ** p<0.01; *** p<0.001).

In PMH this upregulation of *Bsep* gene expression can be already significantly inhibited by pretreatment with IL-1β or TNF-α at concentrations as low as 0.1ng/ml for IL-1β and 0.5 ng/ml for TNF-α, respectively (Fig 2A and 2C). Similarly already concentrations of 0,1 ng/ml IL-1β and 1 ng/ml TNF-α are sufficient to significantly impair CDCA-inducible upregulation of *BSEP* gene expression in the human hepatocyte-like cell line HepaRG cells (Fig 2B and 2D).

### Hepatocytes show high sensitivity towards IL-1β and express a broad target gene pattern of chemokines

**Fig 3:**
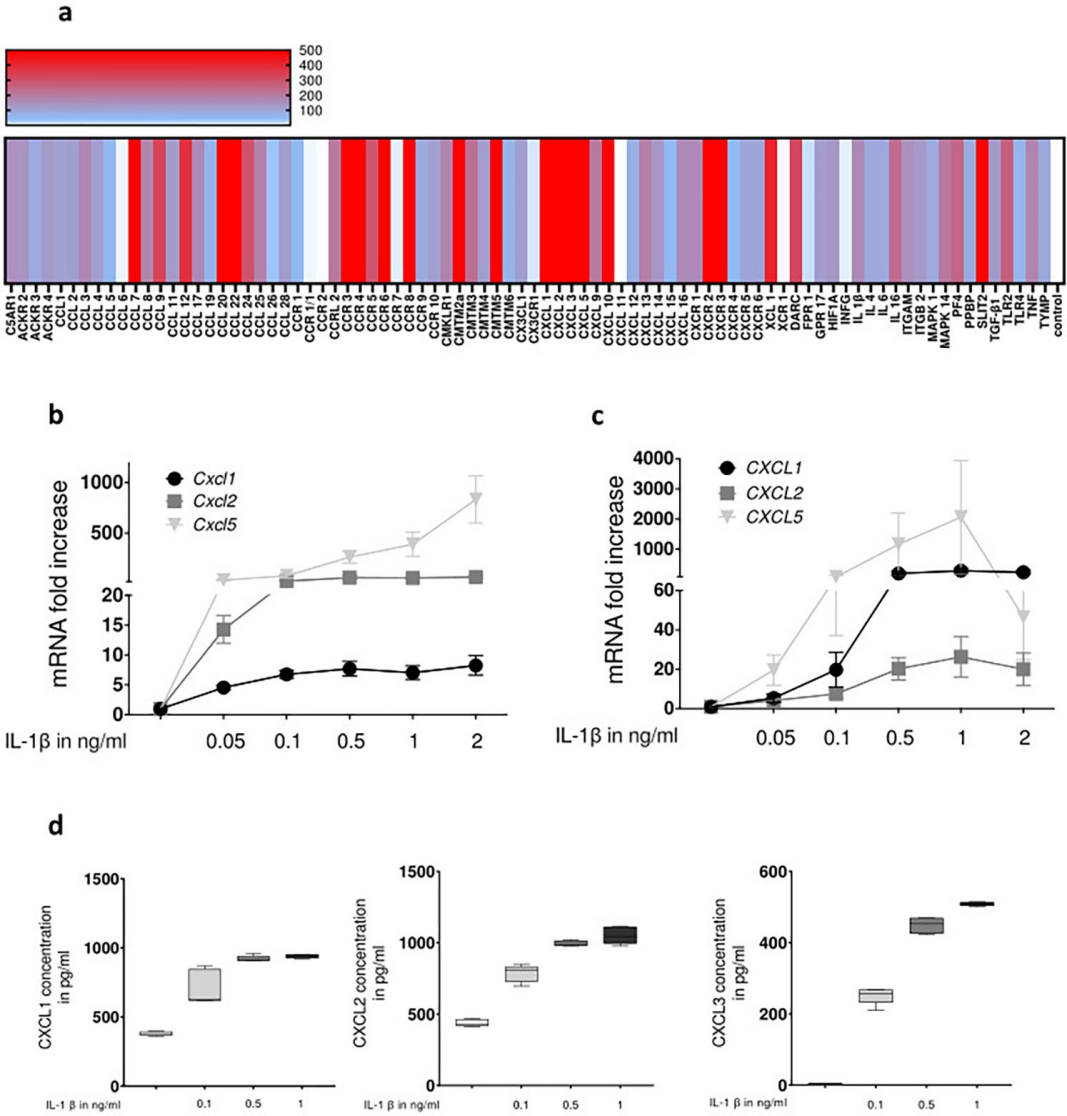
IL-1β enhances the expression of broad pattern of chemokines and concentration dependently upregulates ligands of the CXCR2 receptor at transcript and protein levels. For **a**, **b** and **d** PMH and for **c** HepaRG cells were stimulated with IL-1β for 9 hours with 0.1 ng/ml (**a**) or the concentrations indicated (**b** to **d**). After the respective time period total RNA was prepared (**a** to **c**) or supernatant collected (**d**). For **a**) a PCR based chemokine array was performed according to the manufacturer’s instructions. The colours of the heatmap visualise the relative expression of genes according to the legend in relation to the untreated control. For **b** and **c**) transcript abundance was determined by qRT-PCR using primers specific for CXCL1, 2 and 5 and for **d**) protein concentrations of CXCL1, 2 or 5 was determined using ELISA. Data are presented as box blots based on 3 to 6 replicates. Statistics were calculated by Whitney U test. A p-value of less than 0.05 was considered significant (* p<0.05; ** p<0.01; *** p<0.001).

To identify mediators of potential IL-1β triggered feedback loops the cytokine gene expression of PMH in response to IL-1β was assessed using the RT2 Profiler PCR Array. According to this array IL-1β elicits in hepatocytes the expression of a broad spectrum of cytokines and in particular of chemokines including the expression of respective receptors. Notably this includes several pairs of ligands and the respective receptors such as for the CXCR2 receptor and almost all of its ligands which in mice include CXCL1,2 and CXCL5 (Fig 3A).

**Fig 4:**
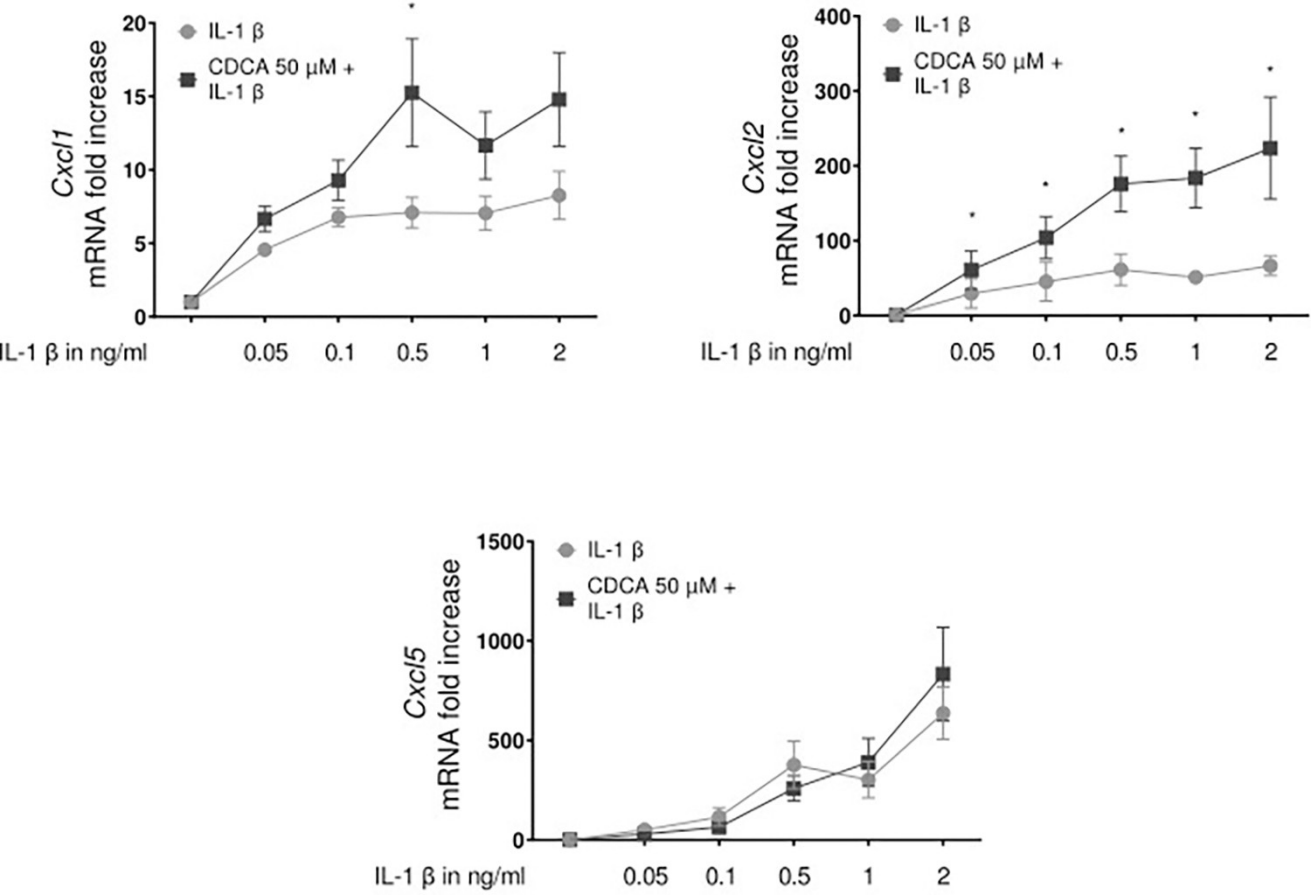
Simultaneous stimulation of PMH with CDCA and IL-1β was ligand-dependently able to further enhance expression. PMH were preincubated with different concentrations of IL-1β for one hour and if indicated followed by stimulation with 50 μM CDCA for 8 hours. Thereafter total RNA was prepared and transcript abundance was determined by qRT-PCR using primers specific for CXCL1, 2 or 5 as indicated. Data are presented as box blots based on 3 to 6 replicates. Statistics were calculated by Whitney U test. A p-value of less than 0.05 was considered significant (* p<0.05; ** p<0.01; *** p<0.001).

Based on these results, a more detailed dose-response analysis of IL-1β -induced expression of CXCL1, 2 and 5 was performed by qPCR (Fig 3B and 3C) and subsequent protein production was determined by ELISA (Fig 3D). As summarized in Fig 3, IL-1β concentrations below 0.1 ng/ml were able to trigger expression of these chemokines in PMH and HepaRG cell lines, with CXCL5 displaying the strongest increase in both cell types. This was also reflected at the level of protein released in the supernatant. Interestingly, stimulation of primary hepatocytes with both unconjugated bile acids and IL-1β was ligand-dependently able to further enhance expression of ligands of CXCR2 (Fig 4).

### CXCL1 and CXCL2 hamper CDCA-induced *Bsep* transcript expression in hepatocytes by CXCR2-dependent pathways

**Fig 5:**
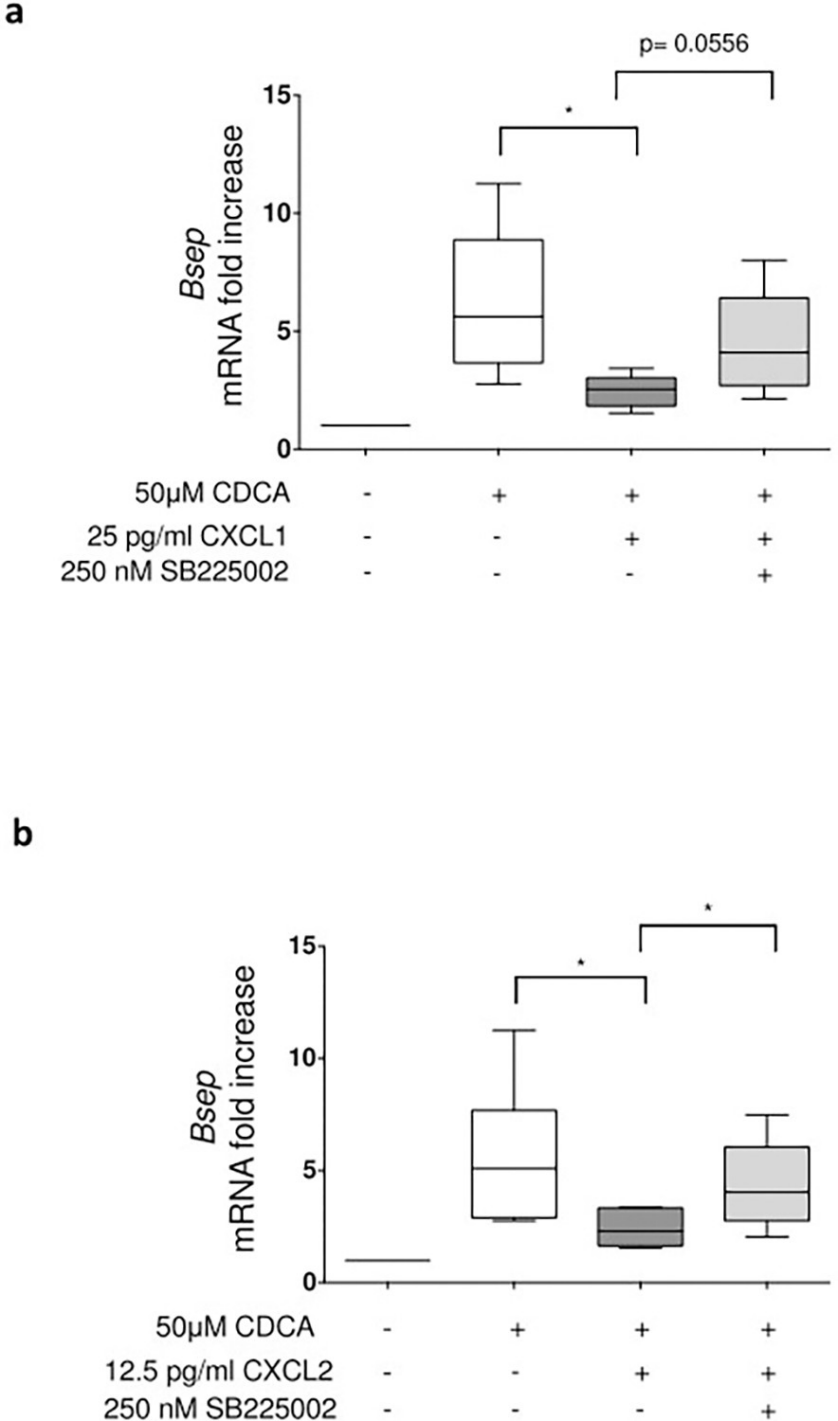
The CXCR2 ligands inhibit CDCA induced *Bsep* gene expression, which is at least partly mediated by CXCR2. For **a** and **b**) PMH cells preincubated for one hour with the 250 nM of the CXCR2 antagonist SB225002 as indicated and thereafter treated with 25 pg/ml CXCL1 or 12,5 pg/ml CXCL2 for 60 minutes. After the respective pretreatment cells were stimulated with 50 μM CDCA for 8 hours. Subsequently RNA was prepared and transcript abundance was determined by qRT-PCR using primers specific for BSEP. Data are presented as box blots based on 5 replicates. Statistics were calculated by Whitney U test. A p-value of less than 0.05 was considered significant (* p<0.05; ** p<0.01; *** p<0.001).

To determine the impact of CXCR2 ligands released in response to IL-1β on CDCA-induced *Bsep* expression, primary murine hepatocytes were co-stimulated with recombinant CXCL1 or 2. Of note, comparable to IL-1β, the application of CXCL1 or CXCL2 was able to inhibit the upregulation of CDCA-induced *Bsep* mRNA expression. This inhibitory effect could be partially reversed by the chemical compound SB225002, which is supposed to specifically block the activation of CXCR2 [37,38], suggesting that activation of this receptor is able to hamper upregulation of *Bsep* expression in response to CDCA (Fig 5).

### IL-1β-mediated inhibition of the *Bsep* gene expression is dependent on CXCR2-controlled pathways

**Fig 6:**
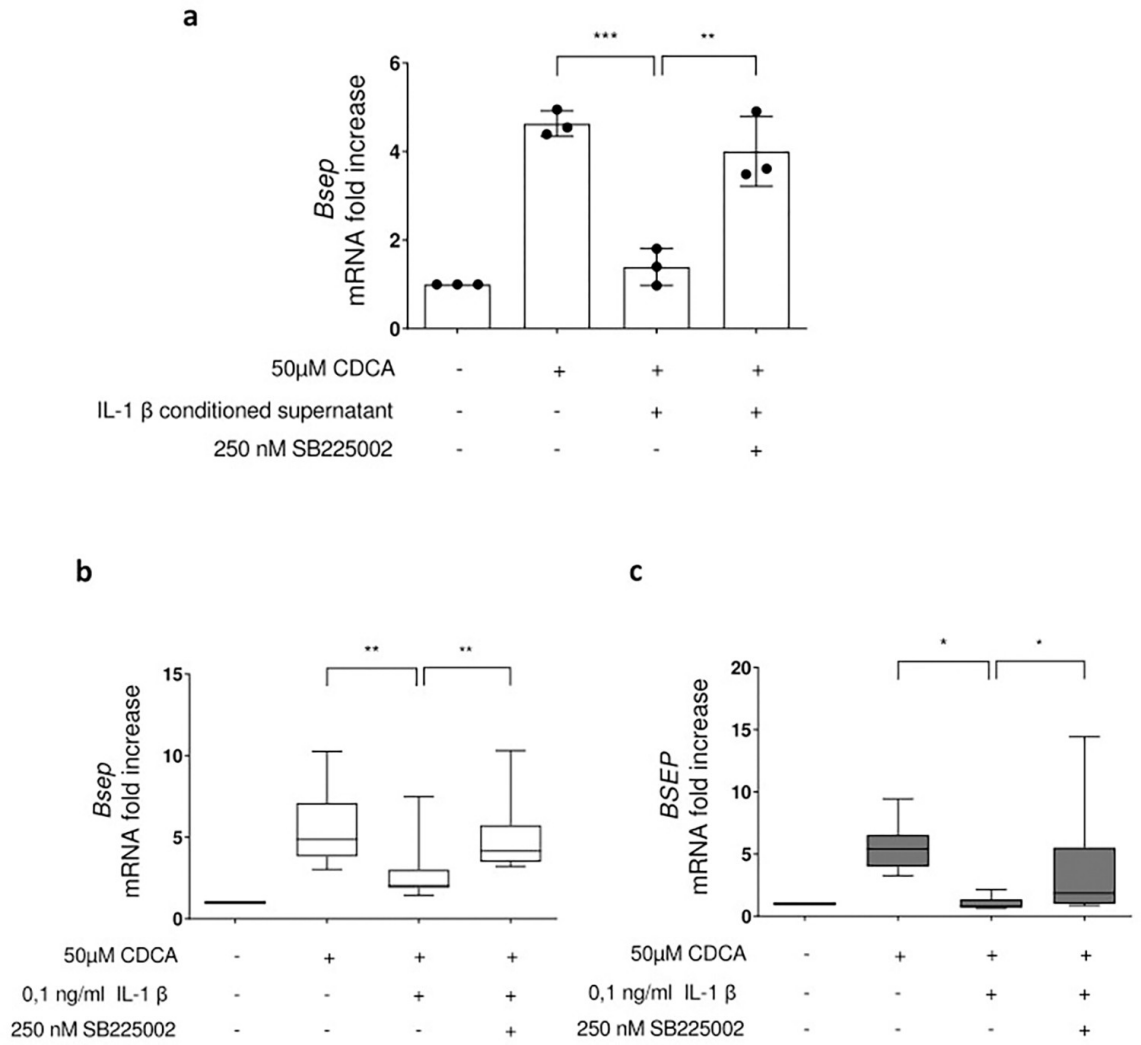
The IL-1β mediated inhibition of the CDCA induced BSEP gene expression is at least partly mediated by CXCR2. For **a**), PMHs were stimulated with 0.1 ng/ml IL-1β for eight hours. The supernatant was then collected and transferred to a new hepatocyte culture, which was treated with 50 μM CDCA after 60 minutes of pre-incubation and incubation was continued for eight hours. For the inhibitor experiments, the PMHs were treated with 250 nM SB 225002 one hour before transfer of the supernatant, which was also supplemented with 250 nM SB 225002. For **b**) PPMHs and **c**) HepaRG cells were preincubated for one hour with the CXCR2 antagonist SB225002 and afterwards stimulated with IL-1β, following the stimulation with CDCA for 8 hours. For **a** to **c**) total RNA was prepared and transcript abundance was determined by qRT-PCR using primers specific for BSEP. Data are presented as box blots based on 5 to 9 replicates. Statistics were calculated by Whitney U test. A p-value of less than 0.05 was considered significant (* p<0.05; ** p<0.01; *** p<0.001).

The above data suggest that CXCR2 ligands are capable of inhibiting CDCA-induced *Bsep* gene expression. To further determine the extent to which IL-1β affects *Bsep* gene expression by inducing a CXCR2 ligand-mediated feedback loop, the influence of inhibition of CXCR2 activation by SB225002 treatment on the inhibitory effect of IL-1β was analysed. Strikingly, SB225002-mediated inhibition of CXCR2 activation was able to block the inhibitory effect of supernatants from IL-1β-conditioned hepatocytes on *Bsep* gene expression in response to CDCA in primary murine hepatocytes (Fig 6A). Consistently, the inhibitory effect of IL-1β on CDCA-inducible *Bsep* mRNA expression was almost completely reversed upon inhibition of CXCR2 using SB225002 in primary murine hepatocytes (Fig 6B) as well as in HepaRG cells (Fig 6C). These data suggest that the inhibitory effect of IL-1β on the upregulation of *Bsep* gene expression in response to unconjugated bile acids is at least in part mediated via activation of CXCR2. In support of this similar results were obtained with alternative chemical compounds such as Cpd 19 and SB265610 which have been reported to specifically inhibit the activation of CXCR2 (S2 Fig in S1 File).

**Fig 7:**
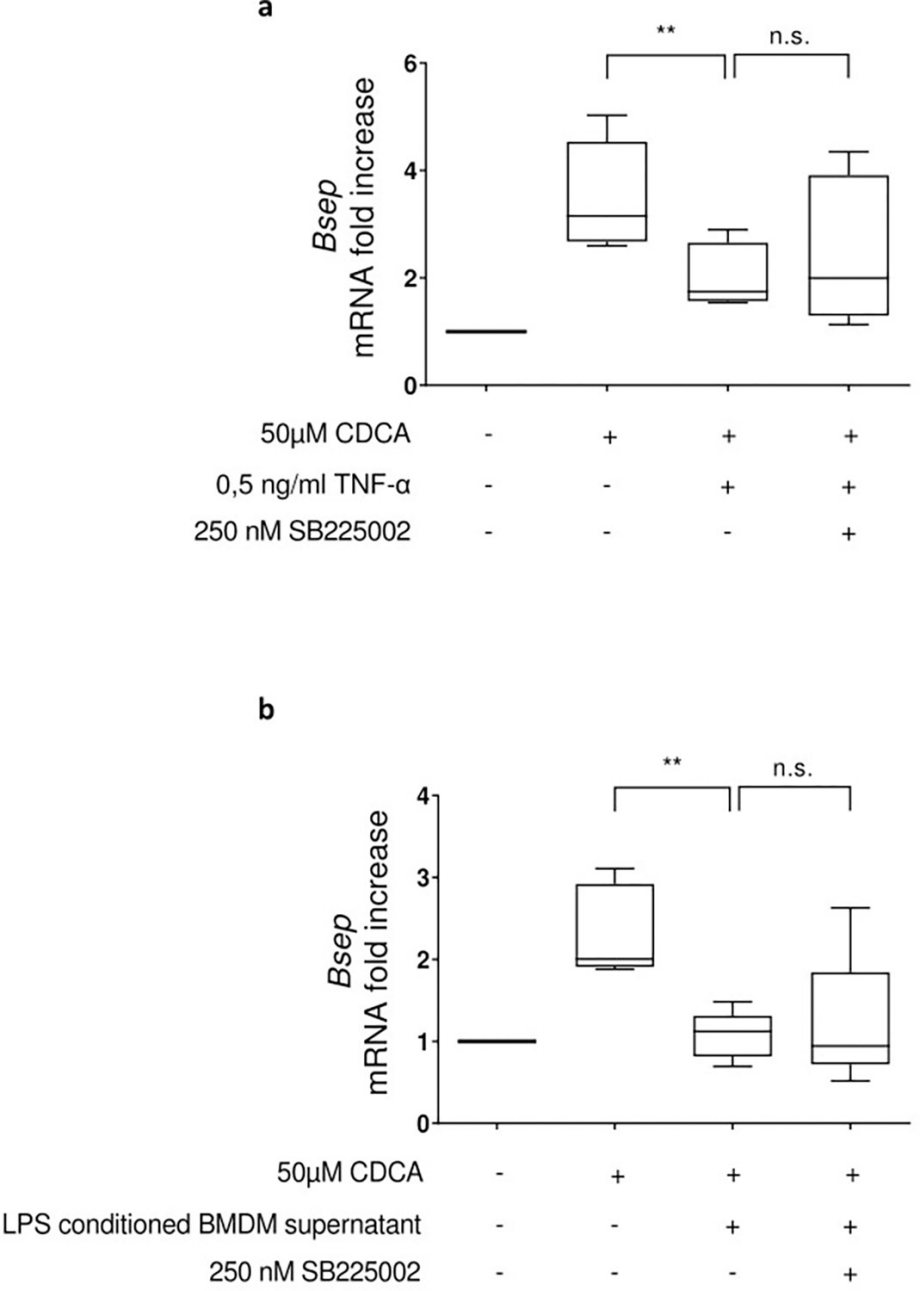
The chemokine receptor antagonist SB 225002 has no significant effect on TNF-α or macrophage secreted cytokine mediated inhibition of CDCA induced *Bsep* mRNA expression in primary mouse hepatocytes. For **a**) PMHs were preincubated for one hour with the CXCR2 antagonist SB225002 and subsequently stimulated with TNF-α, following a stimulation period of 8 hours with CDCA. For **b**) murine BMDM were treated with 10 ng/ml LPS for 10 hours. The supernatant was then collected and transferred to a hepatocyte culture, which was treated with 50 μM CDCA after 60 minutes of pre-incubation and incubation was continued for another eight hours. For the inhibitor experiments, the PMHs were treated with 250 nM SB 225002 one hour before transfer of the supernatant, which was also supplemented with 250 nM SB 225002. For **a** to **c**) total RNA was prepared and transcript abundance was assessed by qRT-PCR using primers specific for BSEP. Data are presented as box blots based on 5–8 replicates. Statistics were calculated by Whitney U test.

As outlined above the inhibition of BSEP expression under septic conditions could be almost completely abrogated by a combined block of TNF-α and IL-1β [13]. Therefore, in PMH the impact of CXCR2 inhibition was also assessed for the inhibition of CDCA-inducible *Bsep* gene expression by either TNF-α treatment (Fig 7A) or by treatment with supernatants from bone marrow-derived macrophages preconditioned with LPS (Fig 7B). Here, both the inhibitory effect of TNF-α and LPS-preconditioned BMDM supernatant on CDCA-induced *Bsep* gene expression in hepatocytes were, at best, tending to be modifiable by inhibition of CXCR2.

Taken together these data indicate that CXCR2-mediated pathways control the inhibitory effects of IL-1β on CDCA-induced *Bsep* gene expression, whereas the respective effects of TNF-α or LPS-preconditioned BMDM supernatants are rather independent of CXCR2.

## Discussion

Inflammation-induced cholestasis primarily arises from disturbances in the expression of bile salt transporters, leading to a significant inhibition of bile salt excretion. In addition to this impaired export mechanism, there is also a dysregulation of the uptake of bile acid in to the hepatocyte from the blood via the sodium taurocholate cotransporting polypeptide (NTCP) [39,40], which has been also reported to be downregulated in the course of sepsis. In addition, there is also evidence that, apart from the dysregulation of bile acid transporter expression in hepatocytes, a downregulation of the apical sodium-dependent bile acid transporter (ASBT) occurs in the enterocyte during an inflammatory response [41,42]. This suggests that the systemic inflammatory response not only disrupts bile acid excretion, but also leads to impairment of the enterohepatic circulation of bile acid. Experimental studies utilizing antagonizing antibodies indicate that the downregulation of bile salt transporter expression in this context is predominantly mediated by pro-inflammatory cytokines, specifically IL-1β and TNF-α [13,43].

By analyzing the relevance of possible feedback loops in mediating the inhibitory effect of these cytokines on the CDCA-inducible upregulation of *Bsep* expression this study provides novel evidence suggesting that at least IL-1β mediates its inhibitory effect on *Bsep* expression through a co-activated CXCR2-dependent pathway that regulates negative feedback control.

For these analysis a culture model of primary hepatocytes was used allowing to examine these cells under steroid-free conditions without undergoing epithelia- to-mesenchymal transition. Additionally, it has been reported that these culture conditions mimic the in vivo effects of hepatocytes in response to various cytokines [43].

FXR-mediated upregulation of *Bsep* expression is mediated in particular by non-conjugated bile acids such as CDCA [26,27,44]. The data provided herein indicate that this FXR-dependent (Fig 1B) upregulation of *Bsep* gene expression in response to CDCA can be almost significantly inhibited by concentrations of as low as 0.1 ng/ml IL-1β or for TNF-α 0.5 ng/ml in murine PMH and 1 ng/ml in HepaRG cells (Fig 2). Thus, these data support the notion of previous studies on the inhibitory influence of the inflammatory cytokines TNF-α and IL-1β on *Bsep* gene expression [43,45]. However, in contrast to the present study, these analyses only investigated the effect of inflammatory cytokines on basal *Bsep* gene expression and not after their upregulation by pretreatment with non-conjugated bile acids. Furthermore, the cytokine concentrations used in the earlier studies were much higher than in the present study, with an IL-1β concentration of 1 ng/ml and a TNF-α concentration of 100 ng/ml.

The data summarized herein further support that hepatocytes are not only target cells for cytokines, but like a wide range of other cell types [46], including non-parenchymal liver cells such as macrophages, sinusoidal endothelial cells or hepatic stellate cells, depending on the stimulus and context, are able to produce relevant amounts of certain immune regulatory mediators, in particular chemokines [47,48]. Thus, in addition to non-parenchymal cells and in particular macrophages [16], they play an active role in the inflammatory response an thus contribute to shape the composition of immune cell populations in the liver by the release of chemotactic signals. This is also evident from the analysis of cytokine expression, with particular reference to chemokines induced by IL-1β in primary hepatocytes, which shows that IL-1β mainly induces increased chemokine expression in these cells. Thereby, in response to IL-1β stimulation, particularly the chemokines of the CXC family are upregulated in hepatocytes, both at the level of gene expression and at the level of protein synthesis. Notably, gene expression of in particular CXCL2 was further enhanced by combined stimulation of hepatocytes with bile acids and IL-1β, highlighting the role of bile acids as DAMPS in the context of sepsis-associated cholestasis [34]. Among others, members of the CXC family that bind to CXCR2 play a key role in inflammation by chemotactically attracting neutrophil granulocytes in particular, thereby significantly influencing the inflammatory response and the resulting tissue damage [49–54]. Consistently, several reports have highlighted the role of CXCR2 ligands in the context of liver-specific pathologies such as obstructive cholestasis [38,55,56]. In this context, the observations summarized here are interesting because they suggest that CXCR2 ligands do not only act as chemotactic signals. They also have a direct regulatory influence on the expression of transport molecules in hepatocytes, thereby promoting cholestasis. Thus, a relevant part of the suppressive effect of IL-1β on the expression of the bile salt transporter BSEP can actually be abolished by a CXCR2-specific inhibitor and can indeed be attributed to the effect of CXCR2 ligands, such as CXCL1, 2, 3 and 5. As far as can be assessed on the basis of the results presented here, only the inhibitory effects of IL-1β on *Bsep* gene expression seems to be mediated by a CXCR2-dependent feedback mechanism, but not the inhibitory effect of TNF-α or the effect of the supernatant from LPS-stimulated macrophages. Consequently, neither the inhibition of CDCA-induced *Bsep* expression by TNF-α nor by the supernatant of LPS-conditioned macrophages can be reversed by inhibition of CXCR2-mediated signaling. The pathophysiological relevance of these findings *in vivo* in the context of inflammation-induced will be the subject of further investigations. However, the fact that cholestasis induced by an LPS-induced inflammatory response can only be attenuated by the combined administration of antagonistic antibodies against both TNF-α as well as IL-1β suggests that IL-1β has a relevant role in this context. Taking these findings into account, it seems likely that the CXCR2-mediated feedback mechanism described here may be relevant at least for the part of inflammation-induced cholestasis mediated by IL-1β.

## Material and methods

### Laboratory animals and husbandry

Wt C57BL/6J mice were purchased from Janvier, Le Genest St. Isle, France. The knock out mice fxr ^-/-^ were provided by Karl Lang, University of Essen maintained on a C57BL/6J background [57].

The experimental animals were kept and bred under specific pathogen-free (SPF) conditions at the animal facility of the University of Duesseldorf. Hygiene monitoring was conducted quarterly in accordance with the recommendations of the Federation of European Laboratory Animal Science Associations for health monitoring in laboratory animal facilities. The animals were kept under a strict day-night cycle (12 h / 12 h) at 22°C and 50% RH and free access to drinking water and standard feed. Male animals aged eight to twelve weeks were used for the experiments. All animal experiments were approved by the Federal Ministry for Nature, Environment and Consumers’ Protection of the state of North Rhine-Westphalia and were performed in accordance to the respective national, federal and institutional regulations (Az. 81–02.04.2017.A406, 84–02.04.2013.A464, 84–02.04.2012.A175). The authors confirm that all experiments were done in accordance to the ARRIVE guidelines.

### Chemicals

IL-1β (human): Roche; IL-1β (murine): Jena Bioscience; Cpd 19: Calbiochem; SB 225002: Tocris Biosciences, TNF α-human (Sigma Aldrich Chemie), TNF α (murin): Roche; LPS (murin): Sigma Aldrich Chemie; CXCL1, CXCL2 (murin): R&D Systems; SB 265610: Sigma Aldrich Chemie. All other chemicals are listed in the Supplementary material and were purchased from Merck and Sigma Aldrich Chemie unless otherwise stated.

### Cell isolation and sandwich culture of primary murine hepatocytes

The isolation and cultivation of the primary mouse hepatocytes was performed according to the method described in detail in [58,59]. Following the administration of ketamine (100 μg per gram of body weight) and xylazine (5 μg per gram of body weight) via intraperitoneal injection, the abdominal cavity of the anaesthetised mouse was opened, cells were isolated by perfusing the liver via the portal vein with an EGTA-containing buffer (HANKS buffer I), followed by a collagenase-containing buffer (HANKS buffer II) to digest the liver tissue (the composition of the buffers is described in the S1 Table in S1 File. The animals die under anesthesia (ketamine and xylazine) due to blood loss resulting from the opening of the vena cava inferior in the course of liver resection The cells were flushed out with William´s medium and the cell suspension filtered through a 70 μm cell strainer. To remove CD11b positive cells (recruited immune cells), the cells were centrifuged for 3 minutes at 50 g and the cell pellet was resuspended in autoMACS rinsing Solution supplemented with 0.5% [w/v] BSA. After renewed centrifugation the cells were resuspended with magnetic coupled antibodies against CD11b (Miltenyi Biotec). After incubation CD11b positive cells were removed when flowing through a magnetic field. The hepatocytes were resuspended in attachement medium (see S1 Table in S1 File). 0.8 x 10^6^ vital hepatocytes were cultivated per well in a 6-well plate in a so called sandwich culture. Here the cells were surrounded by collagen and cultivated with starvation medium. For experiments performed in FXR-KO mice and corresponding WT control (Fig 1B), isolation of hepatocytes from mice was conducted following the administration of carbon dioxide (CO₂) in excess of the recommended dosage, in accordance with the 2020 AVMA Guidelines, the German Animal Welfare Act (TierSchG), and the Ordinance on the Protection of Laboratory Animals (TierSchVersV).

### Cell isolation and sandwich culture of primary murine hepatocytes

The generation of bone marrow-derived macrophages is already established and previously in detail described [59]. The tibia and femur were extracted from mice upon the isolation of hepatocytes. The bone marrow was then rinsed out with a syringe using rinsing medium. Scattered cells were incubated overnight in DMEM (1g/l glucose) supplemented with 1% penicillin/streptomycin. Non-adherent cells were harvested, centrifuged and resuspended in same DMEM medium supplemented with 10 ng/ml macrophage colony-stimulating factor (M-CSF, PeproTech, #315–02). Cells were seeded in 5 plates of 15 cm diameter culture dishes with 20 ml medium supplemented with M-CSF. The cells differentiate into BMDM during seven days of cultivation with gradual increase of the medium.

### Cultivation of HepaRG cells

HepaRG cells were purchased from Life Technologies and kept according to published protocols [60,61]. Shortly, cells were seeded and kept in culture for two weeks (composition of the media is described S1 Table in S1 File). Afterwards, they were splitted and kept in medium supplemented with 2% dimethyl sulfoxide (DMSO) for differentiation in a co-culture of hepatocytes and biliary cells for another two weeks. Experiments were done only with hepatocytes, excluding the biliary cells by short term trypsinization when only hepatocytes detached while biliary cells remain attached.

### RNA isolation and cDNA synthesis

The isolation of murine RNA was done by RNasy Mini Kit (Qiagen) due to manufactors protocol. The isolation of RNA of HepaRG cells was performed by simplyRNA Tissue Kit (Promega). In order to determine the purity and concentration of the RNA, it was examined spectrophotometrically on the nano-drop at an absorbance of 260 nm/280 nm. To obtain complementary DNA (cDNA), 1 μg of RNA per experimental sample was processed with the QuantiTect cDNA Synthesis Kit (Qiagen) according to the manufacturer’s instructions. For subsequent processing by real-time polymerase chain reaction (RT PCR), the cDNA was diluted with nuclease-free water to a concentration of 10 ng/μl.

### Oligonucleotides

The oligonucleotides were purchased from MWG Biotech and were used for quantitative expression analysis by polymerase chain reaction (PCR). According to the manufacturer’s instructions, the oligonucleotides were dissolved (10 pg/μl). For practical reaction application, the corresponding primer pairs were diluted again 1:10 (final concentration 0,4 pg/μl).

For sense and antisense base sequence for the primers of HPRT, BSEP, CXCL1, CXCL2, CXCL3 and CXCL5 see S2 Table in S1 File.

### Quantitative real-time PCR

RT PCR was performed on the ViiA7 RT-PCR system (Applied Biosystems/Thermo Fisher) in 96-hole microtitre plates using the oligonucleotide primers described above. Each reaction batch contained a total volume of 25 μl, which consisted of 1.2 μl cDNA (10 ng/μl), 12.5 μl GoTaq qPCR Master Mix (Promega), 9.3 μl nuclease-free H_2_O and 1 μl each of the corresponding sense and antisense primers (10 pmol/μl). The sequence included a single step of 2 min at 50°C, an initial denaturation at 95°C for 10 min, followed by 40 cycles, each cycle consisting of 15 sec at 95°C (denaturation) and 1 min each at 60°C (annealing and elongation). A melting curve analysis for quality assurance followed each run (15 seconds at 95°C, 1 minute at 60°C, 15 seconds at 95°C). For each condition in an experiment, two ΔCt-values were determined in the RT PCR and the arithmetic mean was formed from these.

### Cytotoxity assay

The CellTiter96® AQueous One Solution Assay (Promega) was performed for cytotoxicity measurement in murine hepatocytes upon 25, 50 and 100 μM CDCA for 8 hours treatment according to the manufacturer’s protocol. The measurement was performed photometrically on the Multiskan plate reader (Thermo Scientific).

### Chemokine profiler assays

The RT2 Profiler PCR Array (Qiagen) was performed using murine hepatocytes stimulated with 0,1 ng/ml IL-1β for 8 hours according to the manufacturer’s instructions and measured and evaluated with the specified settings (measurement with the ViiA7 RT-PCR System, Applied Biosystems).

The Proteom Profiler Mouse Chemokine Array was performed with cell culture supernatant from primary murine hepatocytes stimulated with 0,1 ng/ml IL-1β for 8 hours according to the manufacturer (R&D System). Immunodetection was performed using strepdavidin-HRP solution and X-ray films (BioMax Light-1, Kodak) in a developer machine (Curix 60, AGFA). Densitometric evaluation of the signals obtained from the dot-blot method of the Proteom Profiler Array was performed using ImageJ software (Wayne Rasband). Values were calculated relative to the control.

### Elisa assay

The concentration of the chemokines CXCL 1 (KC), CXCL 2 (MIP-2) and CXCL5 (Lix) in the supernatant of murine hepatocytes after stimulation with 0,1 ng/ml IL-1β for 8 hours was measured by the antibody-based ELISA method according to the manufacturer’s instructions (R&D System). The measurement was performed photometrically at 450 nm (correction wavelength 540 nm) on the Multiskan plate reader (Thermo Scientific).

### Statistical analysis

Data presented in tables or graphs were expressed as mean ± S.E.M. (standard error of mean) or as box and whiskers with minimum to maximum. Statistical analysis and graph creation was performed by program GraphPad Prism 6 for Mac OS X, version 6.0 using unpaired test (t-test or Man-Whitney-U test as indicated). The results were considered significant with a probability of error below 5% (p < 0.05).

## Supporting information

S1 File Angendohr et al Supp material. Supplementary Figure S1: CDCA affects cell viability of primary murine hepatocytes. Supplementary Figure S2: The IL-1β mediated inhibition of the CDCA induced BSEP gene. expression is at least partly mediated by CXCR2. Supplementary table 1: Composition of the respective media and buffers. Supplementary table 2: Primer sequences of indicated primer pairs.
(PDF)

S1 Graphical abstract (PDF)

## Abbreviations

BSEP: Bile Salt Export Pump
IL-1β: Interleukin 1 β
LPS: lipopolysaccharide
TNF-α: tumor necrosis factor-α
CDCA: chenodeoxycholic acid
PFIC: progressive familial intrahepatic cholestasis
FXR: farnesoid X receptor
RXR: retinoid X receptor
PMH: primary murine hepatocytes
BMDM: bone marrow-derived macrophages
CXCR2: CXC receptor 2
CXCL1: C-X-C Motif Chemokine Ligand 1
CXCL2: C-X-C Motif Chemokine Ligand 2
CXCL3: C-X-C Motif Chemokine Ligand 3
CXCL5: C-X-C Motif Chemokine Ligand 5
SPF: specific pathogen-free
WT: wildtype
