# Supplementary material for: Interleukin 1 β suppresses bile acid-induced BSEP expression via a CXCR2-dependent feedback mechanism
Source: PLoS One. 2024 Dec 16;19(12):e0315243. doi: 10.1371/journal.pone.0315243 (PMC11649129; doi:10.1371/journal.pone.0315243)
Supplement: S1 File — Supplementary Figure S1: CDCA affects cell viability of primary murine hepatocytes. Supplementary Figure S2: The IL-1β mediated inhibition of the CDCA induced BSEP gene. expression is at least partly mediated by CXCR2. Supplementary table 1: Composition of the respective media and buffers. Supplementary table 2: Primer sequences of indicated primer pairs. (PDF) [file pone.0315243.s001.pdf]

Supplementary material

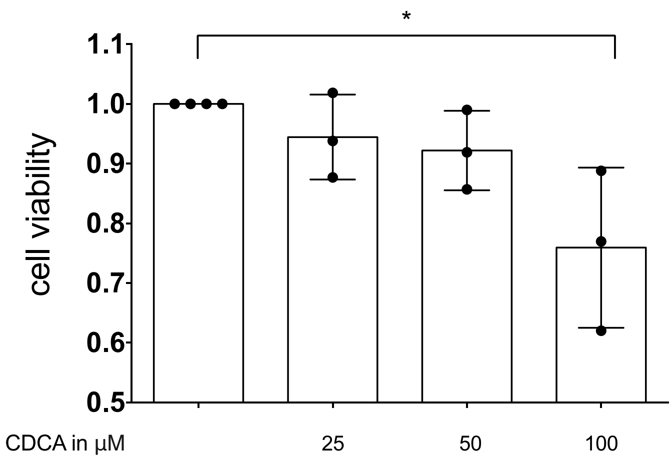

Supplementary Figure S1: CDCA affects cell viability of primary murine hepatocytes
PMH were stimulated for 8 hours with different concentrations of CDCA (25, 50 and 100 µM). The colorimetric detection of formazan formation of stimulated cells compared to untreated controls was determined and served as the basis for percentage detection. Results are expressed as means ± SEM. (n=3)

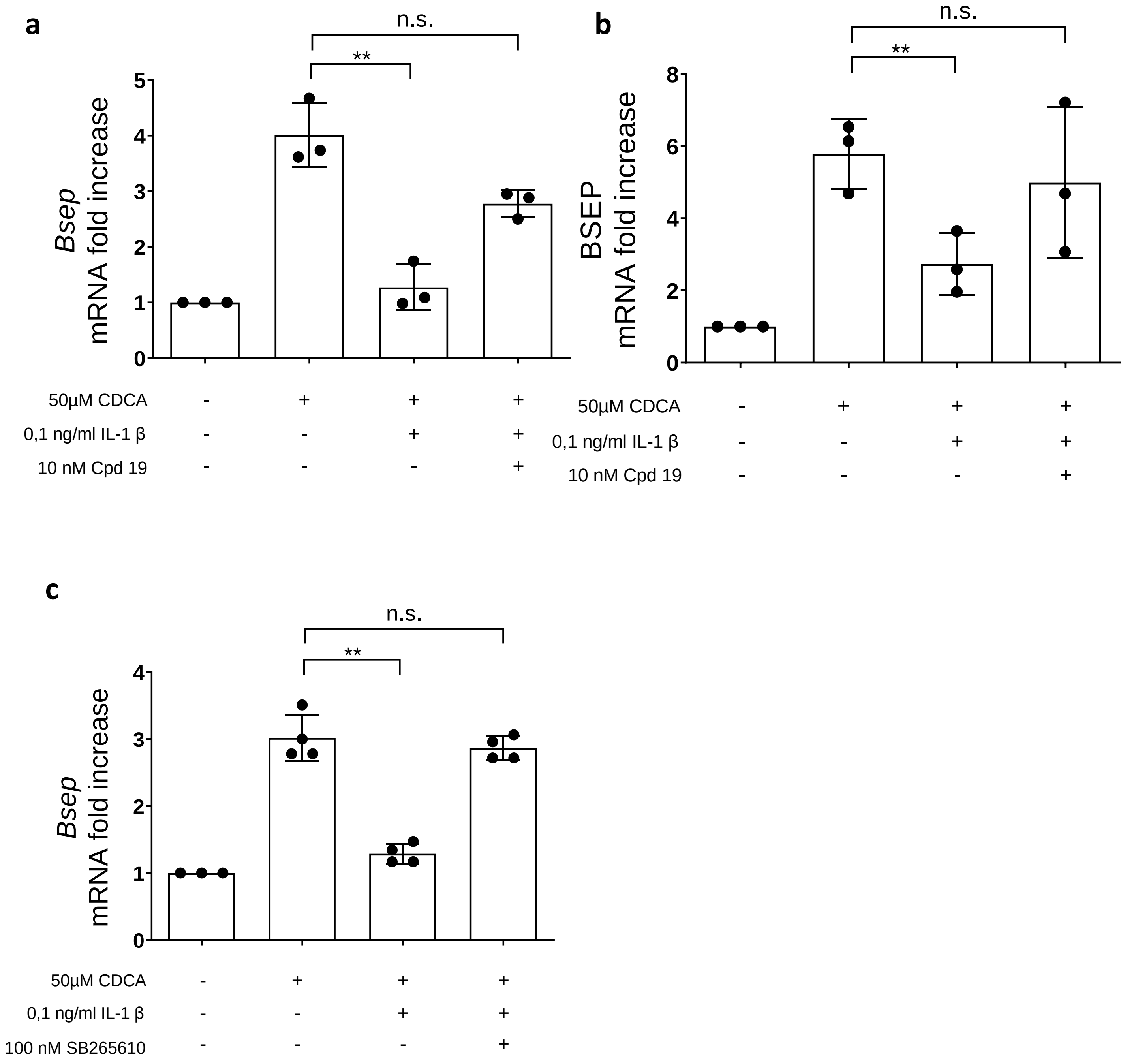

Supplementary Figure S2: The IL-1β mediated inhibition of the CDCA induced BSEP gene expression is at least partly mediated by CXCR2. Primary mouse hepatocytes (a, c) and HepaRG cells (b) were preincubated for one hour with the indicated CXCR2 antagonist, afterwards stimulated with IL-1β, following the stimulation with CDCA for 8 hours. mRNA expression was determined by qRT-PCR.

Results are expressed as means ± SEM. (n=3-4)

Supplementary table 1: Composition of the respective media and buffers

**HANKS buffer**	137 mM (Millimolar)	Natriumchlorid (NaCl)l
	5 nM	Kaliumchlorid (KCl)
	15 mM	Hepes
	0,33 mM	Dinatriumhydrogenphosphat (Na_2HPO_4)
	0,44 mM	Kaliumdihydrogenphosphat (KH_2PO_4)
		pH: 7,4
**buffer Perfusion I**	200 ml	HANKS Puffer
	800 µl (mikroliter)	EGTA 0,5 M
	2 ml	Glucose 10%
**buffer Perfusion II**		HANKS Puffer
	1 ml	$CaCl_2$ (Calciumchlorid) 5 mM
	1 ml	Glucose 10%
	30 mg	Kollagenase
**MACS buffer**	0,5 %	Bovines Serumalbumin (BSA)
	2 mM	EDTA
		PBS
		pH 7,2
**Attachment medium**	500 ml	Williams E Medium
	50 ml	FCS
	1%	Pen/Strep
	2 mM	Glutamin
**Starvation medium**	500 ml	Williams E Medium
	1%	Pen/Strep
	2 mM	Glutamin
**BMDM culture medium**	500 ml	DMEM (1 g/l Glucose)
	10%	FCS
	1%	Pen/Strep (Penicillin/Streptomycin)
	10 ng/ml	M-CSF
**HepaRG medium**	500 ml	Williams E medium
	50 ml	FCS
	1%	Pen/Strep
	2 mM	L-Glutamin
	5 x 10^{-5}M	Hemisuccinat-Hydrocortison
	5 µg/ml	Insulin
**HepaRG differentiation medium**		HepaRG medium
	2%	DMSO

Supplementary table 2: Primer sequences of indicated primer pairs.

**Primer human**	**sense**	**antisense**
**HPRT**	AGGTCGCAAGCTTGCTGG	CCAACACTTCGTGGGGTCC
**BSEP**	ACTAGATGAAGCCACTTCTGCCTTA	TGCACCGTCTTTTCACTTTCTGT
**CXCL1**	AGGGAATTCACCCCAAGAACATC	TCTTAACTATGGGGGATGCAGGA
**CXCL2**	CTTGTCTCAACCCCGCATC	AGGAACAGCCACCAATAAGC
**CXCL5**	CAGACCACGCAAGGAGTTCA	CTTCCCGTTCTTCAGGGAGG
**Primer murin**	sense	antisense
**HPRT**	TATGCCGAGGATTTGGAAAAAGTG	ACAGAGGGCCACAATGTGATG
**BSEP**	TGCTGACAGGATTCGCTTCTC	TTGCTAAGGGCTTCGTTGGT
**CXCL1**	ATGGCTGGGATTCACCTCAAG	AAGCCTCGCGACCATTCTT
**CXCL2**	TGCCAAGGGTGGACTTCAGA	AAGGCAAACTTTTTGACCGC
**CXCL5**	GTTCCATCTCGCCATTCATGC	AAGCAAACACAACGCAGCTC
