# Supplementary figures and images for: Interleukin 1 β suppresses bile acid-induced BSEP expression via a CXCR2-dependent feedback mechanism
Source: PLoS One. 2024 Dec 16;19(12):e0315243. doi: 10.1371/journal.pone.0315243 (PMC11649129; doi:10.1371/journal.pone.0315243)

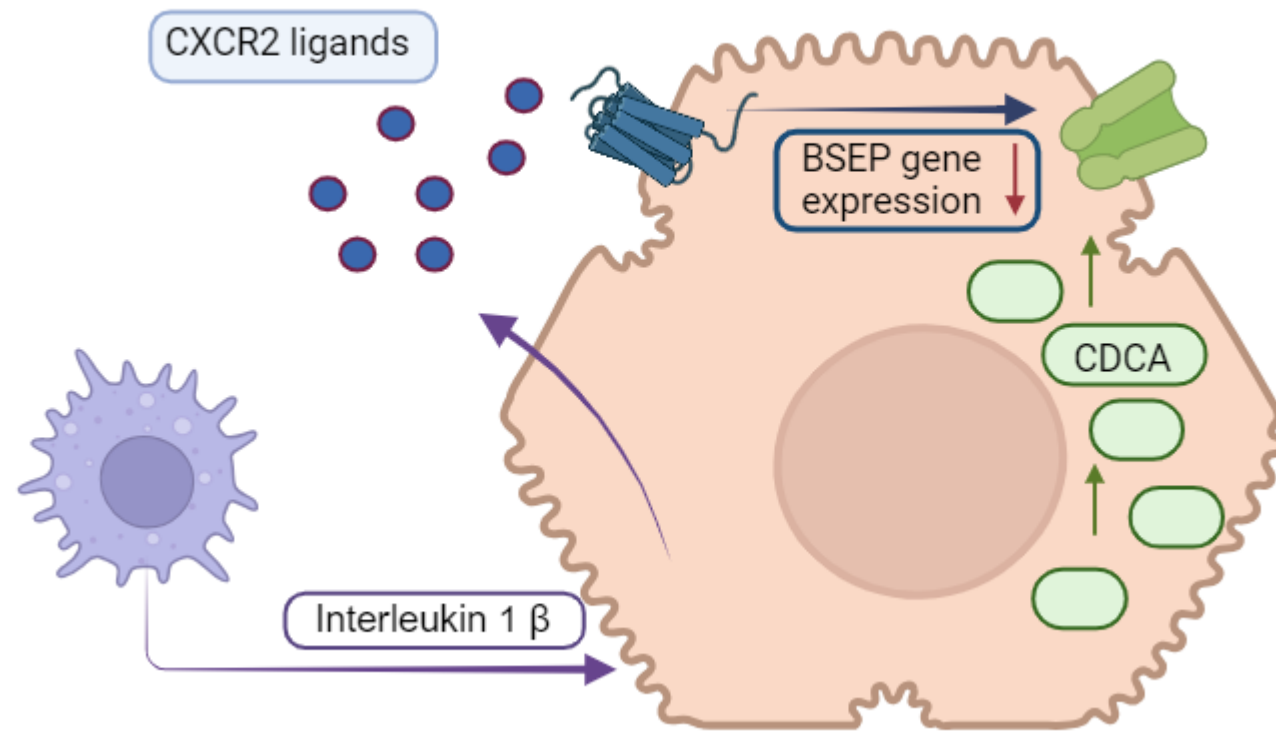

Supplement: S1 Graphical abstract — (PDF) [file pone.0315243.s002.pdf]
